# Supplementary material for: The Effect of Belantamab Mafodotin on Primary Myeloma–Stroma Co-Cultures: Asymmetrical Mitochondrial Transfer between Myeloma Cells and Autologous Bone Marrow Stromal Cells
Source: Int J Mol Sci. 2023 Mar 10;24(6):5303. doi: 10.3390/ijms24065303 (PMC10048929; doi:10.3390/ijms24065303)
Supplement: Supplementary file 1 [file ijms-24-05303-s001.zip › ijms-2225364-supplementary.pdf]

Table S1: Clinical data of multiple myeloma patients participating in the study whose cell cultures showed excellent in vitro growth potential.

Sample ID	Age	Sex	Ig isotype	Primary genetic alteration	Newly diagnosed/Relapsed	Drug exposed	Drug resistant
#108	83	Female	IgA lambda	t(4;14)	Relapsed	Melphalan<br>Prednisolon<br>Bortezomib	Bortezomib<br>Bendamustine<br>Dexamethason
#113	81	Male	IgG lambda	Hyperdiploidy	Newly diagnosed	NA	NA
#118	70	Male	IgG kappa	Hyperdiploidy	Newly diagnosed	NA	NA
#123	85	Female	IgG kappa	t(4;14)	Relapsed	Melphalan<br>Prednisolon<br>Bortezomib	NA
#126	74	Female	IgG lambda	Hyperdiploidy	Relapsed	Vincristine<br>Doxorubicine<br>Dexamethason<br>Melphalan<br>Lenalidomid<br>Bortezomib	Bortezomib<br>Dexamethason
#130	53	Male	IgG kappa	Hyperdiploidy	Newly diagnosed	NA	NA
#132	56	Male	IgA kappa	t(4;14)	Newly diagnosed	NA	NA
#138	78	Female	IgG kappa	t(11;14)	Newly diagnosed	NA	NA
#139	75	Male	IgG kappa	t(11;14)	Relapsed	Bortezomib<br>Dexamethason	NA
#165	77	Female	IgA kappa	t(11;14)	Newly diagnosed	NA	NA
